# Supplementary material for: Enhancing Men’s Awareness of Testicular Diseases (E-MAT) using virtual reality: A randomised pilot feasibility study and mixed method process evaluation
Source: PLoS One. 2024 Jul 22;19(7):e0307426. doi: 10.1371/journal.pone.0307426 (PMC11262699; doi:10.1371/journal.pone.0307426)
Supplement: S1 File — (DOCX) [file pone.0307426.s001.docx]

**ENHANCING MEN’S AWARENESS OF TESTICULAR DISEASES (E-MAT):
A RESEARCH STUDY**

**HRB DIFA-2020-028**

**Study Protocol**

**1. Enhancing Men’s Awareness of Testicular Diseases (E-MAT): A Randomised Feasibility Trial**

**1.1 Aim:** In order to determine the processes needed to conduct a definitive trial, identify unforeseen problems, and plan for progression to a definitive trial, we will test the effect of E-MAT_VR_ (intervention) compared to E-MAT_E_ (control) on primary outcomes (testicular knowledge and self- examination behaviours) and secondary outcomes (testicular awareness, frequency of self-examination, recommendation of self-examination to others, help-seeking intentions, implementation intentions, and perceived risk) among GAA players and coaches. Outcomes (all self-reported) will be measured at baseline (T0), immediately post-test (T1), and three months post-test (T2) using electronic surveys.

**1.2 Inclusion criteria**

1. Biological males***.***
2. Members of the target GAA clubs since testicular injuries are common in field sports like hurling.
3. Residing in Ireland.
4. Aged 18-50 years (age group at risk for testicular diseases).

**1.3 Exclusion criteria**

1. History of seizures.
2. History of motion sickness.

**1.4 Setting**

Several GAA clubs are adopting “The Healthy Club” philosophy to improve the health of their members (https://www.gaa.ie/my-gaa/community-and-health/healthy-club/). Data will be collected in six geographically dispersed GAA clubs.

**1.5 Primary outcomes**

1. Testicular knowledge (Dichotomous)
2. Testicular self-examination behaviours (Dichotomous)

**1.6 Secondary outcomes**

1. Testicular awareness (Likert scale)
2. Frequency of testicular self-examination (Likert scale)
3. Recommendation of testicular self-examination to others (Likert scale)
4. Help-seeking intentions (Likert scale)
5. Implementation intentions (Likert scale)
6. Perceived risk of testicular disorders (Likert scale)

Outcomes will be measured at baseline (T0), immediately post-test (T1), and three months post-test (T2) using the Castor EDC software (https://www.castoredc.com/).

A Research Assistant and/or Research Support Officer will be present to ensure participant safety. There is no in-person follow-up. Participants will be contacted (phone call/text message/email) to ensure completion of outcome measurement at T2. Non-respondents and those who withdraw will be considered lost to follow-up. Retention is key to ensuring power and internal validity. This will be addressed in the SWAT described below.

**1.7 Sample size**

Given that the goal of a feasibility trial is to identify problems that would impede the conduct of a larger, definitive efficacy trial, we have set the sample size at 59 based on advice from [Viechtbauer et al. (2015)](https://pubmed.ncbi.nlm.nih.gov/26146089/) which is aimed at being able to detect failures in study processes that would occur just 5% of the time (with 95% confidence). Bearing in mind a potential attrition rate of 25-30%, 75-80 participants will be recruited.

**1.8 Randomisation and allocation concealment**

For each of the six participating GAA clubs, individual participants will be randomised with the same probability to one of the two arms (E-MAT_VR_/E-MAT_E_). Allocation concealment will be maintained using a computerised system. Once participants have consented and their baseline assessment has been entered into the computer database, their allocation will be given by automated email. Randomisation and allocation concealment will be conducted according to the standard operating procedures (SOPs) of the Statistics and Data Analysis Unit of the HRB Clinical Research Facility-Cork (HRB CRF-C).

**1.9 Blinding**

Given the differences between the two arms, participants will be aware of the arm they have been randomised into. Outcome assessments will be self-reported and returned to research staff with no information on allocation. Blinding will be maintained throughout data analysis. Unblinding will be facilitated by the independent statistician following completion of the protocol-specified data analysis.

**1.10 Data analysis**

Analyses will be conducted by the lead statistician, under the SOPs of the Statistics and Data Analysis Unit of the HRB CRF-C. Before recruitment, a detailed Statistical Analysis Plan will be uploaded alongside the study protocol to the relevant registries. Data will undergo extensive quality checking and potential errors will be verified from source data *a priori.*

Analyses of outcomes will be conducted on an intention-to-treat basis. Differences in outcomes by study arm, over time, will be estimated using generalised linear mixed effects models with the appropriate link function (based on the distribution of the outcome or expected error distribution). We will report minimally adjusted models, which will include effects of intervention arm and time. We will report a fully adjusted model that includes the key predictor variables collected at baseline. Missing data will be evaluated and addressed using appropriate methods (e.g., multiple imputation or inverse-probability weighting). Effect estimates for all pre-specified primary and secondary endpoints, will be publicly reported with 95%CI and exact p-values, in accordance with recent guidance from the American Statistical Association [19].

Analyses will be conducted using transparent, open-science tools. All analysis steps, including cleaning or modifications to study database, will be fully scripted and replicable. Scripts for the key analyses will undergo testing prior to data analysis. Upon completion, the study database will be prepared according to FAIR data principles and be made available along-side the analysis scripts.

**1.11 Ethical considerations**

This study will be conducted in line with ethical principles depicted in the Declaration of Helsinki. Ethical approval will be sought from the Clinical Research Ethics Committee at UCC. Participants will be provided with an information leaflet and will sign informed consent.

Participants will be asked to check with their General Practitioner for abnormalities (e.g., lump) identified during testicular self-examination. They will also be asked to visit the emergency department for sudden and severe testicular pain which is often associated with emergencies such as testicular torsion.

This is a minimal risk study. Violent VR gaming can cause motion sickness and seizures. This is not the case in E-MAT_VR_ which is an educational intervention. However, in order to maintain the safety of participants, those with a history of severe motion sickness and seizures will not be eligible for inclusion.

**2. Mixed-Method Process Evaluation**

**2.1 Objectives**

1. Understand and mitigate potential sources of intervention failure in terms of contextual effects, inputs, engagement, activities, and outcomes.
2. Explore participants’ experiences of E-MAT_VR_ and E-MAT_E_ focusing on perceived effectiveness; feasibility of study and procedures; relations between implementation, mechanisms, and context; and barriers and facilitators to conducting a definitive trial.

**2.2 Design**

This descriptive mixed-method realist evaluation will address “what works, for whom, under what circumstances, and will be informed by MRC guidance. The LOGIC model will form a framework to systematically and visually present, map, and share our understanding of the relationships among resources used to underpin the intervention; activities planned (intervention inputs, processes, actions); outputs; anticipated and unanticipated outcomes; and impact. This will involve observational intervention fidelity checks, dose and reach considerations, completion of survey questions to explore participants’ experiences, and qualitative interviews.

**2.3 Planned activities: Fidelity**

Fidelity checks will explore whether the intervention is being delivered as intended and assess the quality of intervention delivery. Two collaborators will attend intervention delivery to observe and record fidelity to the agreed protocol delivery methods. Observations will be semi-structured and include recording (tick box) pre-specified items (e.g., participants’ behaviour, interest, and engagement) alongside open qualitative observations.

**2.4 Planned activities: Dose**

Time taken to deliver the intervention (dose exposure) and units of intervention completed (dose completeness), participant satisfaction with intervention, interactions with research staff (dose satisfaction), and extent to which participants are receptive to the interventions (dose exposure) will be collected.

**2.5 Impact: Reach**

Proportion of the intended population that participates in the intervention; measured by number of attendees and includes exploration of barriers to participation.

**2.6 Anticipated and unanticipated outcomes/Impact: Survey**

Participants will complete an online survey about their satisfaction (Likert scale) and experiences (open-ended) of participating. Other data that will be collected include timing and difficulties experienced with interventions, frequency of adverse events, and implementation problems.

**2.7 Anticipated/unanticipated outcomes: Interview**

All participants, including non-completers, and the research team involved in data collection will be invited to partake in audio-recorded face-to-face/telephone interviews/focus groups following engagement with E-MAT_VR_/E-MAT_E_. We aim to interview approximately 20 participants. Semi-structured interview/focus group guides will be used for each stakeholder group to guide in-depth discussions. The interview/focus group will focus on the overall experience of the interventions and barriers and facilitators encountered. The interviewer will explore mechanisms through which the intervention/control bring about changes in testicular awareness, which will inform how the effects of the intervention occurred and how these effects might be replicated in a future definitive trial.

**2.8 Context**

Context includes anything peripheral to the intervention that may influence its implementation, reach, or effects. Understanding the context is critical to understanding the potential impact of context on the implementation of the intervention. This will be measured in the survey and interviews discussed earlier.

**2.9 Analyses**

**2.9.1 Quantitative data:** Will be analysed using SPSS and summary descriptive statistics by allocation arm will be presented.

**2.9.2 Qualitative data:** Interviews/focus groups will be digitally recorded, transcribed verbatim, and analysed using qualitative content analysis. Results will be cross-checked by team members to enhance trustworthiness. Data analysis will be iterative such that early interviews can inform questions in later interviews.

Quantitative and qualitative data will be integrated through a narrative synthesis to elicit the process of implementing the intervention. The intervention delivery and what process effects were observed will identify explanatory ‘Context + Mechanism → Process effect’ alignments that explain how the intervention/overall study was perceived, if/why this varied, and how these perceptions affected intervention receptivity.
