# Supplementary material for: Enhancing Men’s Awareness of Testicular Diseases (E-MAT) using virtual reality: A randomised pilot feasibility study and mixed method process evaluation
Source: PLoS One. 2024 Jul 22;19(7):e0307426. doi: 10.1371/journal.pone.0307426 (PMC11262699; doi:10.1371/journal.pone.0307426)
Supplement: S3 File — (DOCX) [file pone.0307426.s003.docx]

**Enhancing Men's Awareness of Testicular Diseases (E-MAT) Using Virtual Reality: A Randomised Pilot Feasibility Study and Mixed Method Process Evaluation**

**Supplementary Tables S1-S11**

**Contents**

[**S1 Table.** Semi-structured interview guide for participants and research personnel 2](#_Toc170206441)

[**S2 Table.**Intervention usability responses at T1 by trial arm 4](#_Toc170206442)

[**S3 Table.** Satisfaction at T1 by trial arm 8](#_Toc170206443)

[**S4 Table.** Participants’ responses to open-ended questions 9](#_Toc170206444)

[**S5 Table.** Testicular self-examination behaviours at baseline 11](#_Toc170206445)

[**S6 Table.**Testicular self-examination behaviours at T2 12](#_Toc170206446)

[**S7 Table.** Reliabilities for multi-item scales 13](#_Toc170206447)

[**S8 Table.**Distribution of testicular awareness scores by trial arm and time point 14](#_Toc170206448)

[**S9 Table.** Perceived risk at by trial arm and time point 15](#_Toc170206449)

[**S10 Table.** Implementation intentions by trial arm and time point 16](#_Toc170206450)

[**S11 Table.** Distribution of General Help Seeking Questionnaire scores by trial arm and time point 18](#_Toc170206451)

# **S1 Table.** Semi-structured interview guide for participants and research personnel

| **Participants** | **Questions** |
| --- | --- |
| **E-MAT_VR_ participants** | 1. Can you talk to me about your overall personal experience of participating in the E-MAT study? 2. What were the main elements of the E-MAT_VR_ intervention which worked best for you? Why? 3. What were the main elements of the E-MAT_VR_ intervention which did not work well for you? Why? (Probe: what did you like least about the E-MAT_VR_ intervention?) 4. In terms of the timing of the E-MAT_VR_ intervention, how ideally should it be offered to men? 5. What were your experiences of using the E-MAT_VR_ intervention? (Probe: length of time for completion, potential burden in terms of usability) 6. Is there anything you would change about the current E-MAT_VR_ intervention, based on your personal experience? 7. If you could influence the design and planning for future VR interventions in the context of health promotion, what would they look like? |
| **E-MAT_E_ participants** | 1. Can you talk to me about your overall personal experience of participating in the E-MAT study? 2. What were the main elements of the E-MAT intervention using PDF reader on a tablet which worked best for you? Why? 3. What were the main elements of the E-MAT intervention using PDF reader on a tablet which did not work well for you? Why? (Probe: what did you like least about the intervention?) 4. In terms of the time it took to complete the E-MAT intervention using PDF reader on a tablet, how ideally should it be offered to men? 5. What were your experiences of using the E-MAT intervention using PDF reader on a tablet? (Probes: length of time for completion, potential burden in terms of usability) 6. Is there anything you would change about the E-MAT intervention using PDF reader on a tablet, based on your personal experience? 7. If you could influence the design and planning for interventions in the context of health promotion, what would they look like? |
| **Research personnel** | 1. Can you talk to me about your overall personal experience of participating in the study? 2. Can you briefly describe your role in relation to the study? Please outline any issues/concerns you had in relation to the study? (Probe: practical issues such as resources, training, administration, and IT supports etc.) 3. In your opinion, was the intervention delivered as originally intended? (Probe: if not, please describe the main adaptations made to suit the local practice context) 4. What is your perspective on the use of the E-MAT_VR_ intervention? 5. What were the main components of the intervention which worked best in terms of facilitating implementation of the VR intervention? 6. What were the main components of the intervention which did not work well in terms of facilitating implementation of the VR intervention? (Probes: workload, practicability, resources/costs, time required, accrual/retention challenges, unintended outcomes) 7. Do you have any proposals in terms of possible solutions to problems encountered with regards to implementing the intervention? (Probes: looking at wider and local context of the intervention implementation) 8. What are your reflections on issues of nonparticipation and participant drop-outs from the study, in terms of reasons why and what could be done to improve study accrual and attrition rates in future studies? (Probe: uptake) 9. Is there anything you would like to change about the intervention? If yes, what would you change and why? 10. If you could influence the design and planning for future VR interventions in the context of health promotion, what are your views as to what those would entail? (Probe: sustainability, practicality) |

# **S2 Table.**Intervention usability responses at T1 by trial arm

| **Characteristic** | **N** | **E-MAT_E_**, N = 37*^1^* | **E-MAT_VR_**, N = 37*^1^* | **p-value***^2^* |
| --- | --- | --- | --- | --- |
| **The intervention is applicable to men from different ethnic/cultural backgrounds** | 74 |  |  | 0.37 |
| Strongly Disagree |  | 0 (0%) | 0 (0%) |  |
| Disagree |  | 0 (0%) | 0 (0%) |  |
| Neither Agree nor Disagree |  | 1 (2.7%) | 4 (10.8%) |  |
| Agree |  | 22 (59.5%) | 13 (35.1%) |  |
| Strongly Agree |  | 14 (37.8%) | 20 (54.1%) |  |
| **The intervention is close and applicable to real life** | 74 |  |  | 0.37 |
| Strongly Disagree |  | 0 (0%) | 0 (0%) |  |
| Disagree |  | 0 (0%) | 0 (0%) |  |
| Neither Agree nor Disagree |  | 1 (2.7%) | 1 (2.7%) |  |
| Agree |  | 21 (56.8%) | 17 (45.9%) |  |
| Strongly Agree |  | 15 (40.5%) | 19 (51.4%) |  |
| **I learned something valuable from this intervention** | 74 |  |  | 0.80 |
| Strongly Disagree |  | 0 (0%) | 0 (0%) |  |
| Disagree |  | 0 (0%) | 0 (0%) |  |
| Neither Agree nor Disagree |  | 0 (0%) | 0 (0%) |  |
| Agree |  | 11 (29.7%) | 12 (32.4%) |  |
| Strongly Agree |  | 26 (70.3%) | 25 (67.6%) |  |
| **The device was comfortable** | 74 |  |  | 0.90 |
| Strongly Disagree |  | 1 (2.7%) | 0 (0%) |  |
| Disagree |  | 1 (2.7%) | 3 (8.1%) |  |
| Neither Agree nor Disagree |  | 2 (5.4%) | 0 (0%) |  |
| Agree |  | 12 (32.4%) | 14 (37.8%) |  |
| Strongly Agree |  | 21 (56.8%) | 20 (54.1%) |  |
| **The device was easy to use** | 74 |  |  | 0.20 |
| Strongly Disagree |  | 0 (0%) | 0 (0%) |  |
| Disagree |  | 1 (2.7%) | 0 (0%) |  |
| Neither Agree nor Disagree |  | 3 (8.1%) | 0 (0%) |  |
| Agree |  | 10 (27.0%) | 22 (59.5%) |  |
| Strongly Agree |  | 23 (62.2%) | 15 (40.5%) |  |
| **I was engaged to learn by the device** | 74 |  |  | <0.001 |
| Strongly Disagree |  | 0 (0%) | 0 (0%) |  |
| Disagree |  | 1 (2.7%) | 0 (0%) |  |
| Neither Agree nor Disagree |  | 3 (8.1%) | 0 (0%) |  |
| Agree |  | 22 (59.5%) | 11 (29.7%) |  |
| Strongly Agree |  | 11 (29.7%) | 26 (70.3%) |  |
| **The information provided by the device was clear** | 74 |  |  | 0.062 |
| Strongly Disagree |  | 0 (0%) | 0 (0%) |  |
| Disagree |  | 0 (0%) | 0 (0%) |  |
| Neither Agree nor Disagree |  | 0 (0%) | 0 (0%) |  |
| Agree |  | 20 (54.1%) | 12 (32.4%) |  |
| Strongly Agree |  | 17 (45.9%) | 25 (67.6%) |  |
| **The time it took was reasonable** | 74 |  |  | 0.33 |
| Strongly Disagree |  | 0 (0%) | 0 (0%) |  |
| Disagree |  | 0 (0%) | 0 (0%) |  |
| Neither Agree nor Disagree |  | 0 (0%) | 3 (8.1%) |  |
| Agree |  | 21 (56.8%) | 12 (32.4%) |  |
| Strongly Agree |  | 16 (43.2%) | 22 (59.5%) |  |
| **The intervention is applicable to men who are 18 to 50 years of age** | 74 |  |  | 0.42 |
| Strongly Disagree |  | 0 (0%) | 0 (0%) |  |
| Disagree |  | 0 (0%) | 0 (0%) |  |
| Neither Agree nor Disagree |  | 1 (2.7%) | 0 (0%) |  |
| Agree |  | 20 (54.1%) | 18 (48.6%) |  |
| Strongly Agree |  | 16 (43.2%) | 19 (51.4%) |  |
| *^1^* n (%)  *^2^* Kruskal-Wallis rank sum test | | | | |

# **S3 Table.** Satisfaction at T1 by trial arm

| **Characteristic** | **N** | **E-MAT_E_**, N = 37*^1^* | **E-MAT_VR_**, N = 37*^1^* | **p-value***^2^* |
| --- | --- | --- | --- | --- |
| **My experience using the device to learn about testicular diseases** | 74 |  |  | 0.59 |
| Extremely Dissatisfied |  | 0 (0%) | 0 (0%) |  |
| Dissatisfied |  | 0 (0%) | 0 (0%) |  |
| Neither Satisfied nor Dissatisfied |  | 0 (0%) | 0 (0%) |  |
| Somewhat Satisfied |  | 10 (27.0%) | 8 (21.6%) |  |
| Extremely Satisfied |  | 27 (73.0%) | 29 (78.4%) |  |
| **My overall experience of participating in this study** | 74 |  |  | 0.96 |
| Extremely Dissatisfied |  | 0 (0%) | 0 (0%) |  |
| Dissatisfied |  | 0 (0%) | 0 (0%) |  |
| Neither Satisfied nor Dissatisfied |  | 1 (2.7%) | 0 (0%) |  |
| Somewhat Satisfied |  | 6 (16.2%) | 7 (18.9%) |  |
| Extremely Satisfied |  | 30 (81.1%) | 30 (81.1%) |  |
| *^1^* n (%) | | | | |
| *^2^* Kruskal-Wallis rank sum test | | | | |

# **S4 Table.** Participants’ responses to open-ended questions

| **Categories** | **Term or phrase used** | **n*^1^*** |
| --- | --- | --- |
| Elements of the tablet liked | Easy/ simple/ user-friendly | 22 |
|  | None | 10 |
|  | Quick/ fast | 6 |
|  | Pictures/ images | 4 |
|  | Informative | 3 |
|  | Touch screen | 2 |
|  | Own speed/pace | 2 |
|  | Light, tactile, bright | 1 |
|  | Access | 1 |
|  | Clear slides | 1 |
|  | Handheld | 1 |
| Elements of the tablet not liked | None | 24 |
|  | tablet touchscreen function | 7 |
|  | Length of survey/ slides | 2 |
|  | Repetition | 1 |
|  | Lack of interaction | 1 |
|  | Text heavy | 1 |
|  | Should of worn glasses | 1 |
| Elements of the tablet recommended to change | None | 32 |
|  | Click on a number in bullet form list | 1 |
|  | Make an app for the questionnaire and pdf | 1 |
|  | Perhaps break down the sections / PDF based on the order of the questions asked in the initial questionnaire - pain etc | 1 |
|  | Images were good, could include more. Less words per slide | 1 |
|  | Add video clips maybe | 1 |
| Elements of the VR game liked | Interactive/ engaging | 16 |
|  | Easy | 13 |
|  | Fun/ enjoyable | 7 |
|  | Information | 6 |
|  | ‘Real life’ | 4 |
|  | Humour | 2 |
|  | ‘Irish twist’ | 1 |
|  | Control yourself | 1 |
|  | Being able to Visualise some of the problems with testicles | 1 |
|  | Quick | 1 |
|  | Different way of learning | 1 |
|  | Novelty | 1 |
|  | None | 1 |
| Elements of the VR game not liked | None | 20 |
|  | Nausea/ sickness | 6 |
|  | Dizzy/ disorientating | 5 |
|  | Navigation | 4 |
|  | ‘I think I would go through the game again. My first time I found myself more focused on the VR experience than the content’ | 1 |
|  | ‘The feeling at the start, was fine after a minute when I got used to it’ | 1 |
| Elements of the VR game recommended to change | None | 30 |
|  | More interaction | 2 |
|  | More content | 1 |
|  | Quicker to use | 1 |
|  | Easier to move | 1 |
|  | Maybe better graphics wouldn’t make me as dizzy | 1 |
|  | Make the steering’s somewhat more medical | 1 |

*^1^* Participants were allowed to type multiple responses. n corresponds to the number of times a statement appeared as opposed to the number of participants.

# **S5 Table.** Testicular self-examination behaviours at baseline

| **Characteristic** | **N** | **E-MAT_E_**, N = 37*^1^* | **E-MAT_VR_**, N = 37*^1^* | **Odds ratio (95% CI)***^2^* | **p-value** |
| --- | --- | --- | --- | --- | --- |
| Have you purposefully felt/examined your testicles within the past month? | 74 |  |  | 3.7 (1.7 to 10.2) | 0.01 |
| No |  | 28 (75.7%) | 17 (45.9%) |  |  |
| Yes |  | 9 (24.3%) | 20 (54.1%) |  |  |
| Have you purposefully felt/examined your testicles within the past year? | 74 |  |  | 1.5 (0.5 to 4.5) | 0.43 |
| No |  | 11 (29.7%) | 8 (21.6%) |  |  |
| Yes |  | 26 (70.3%) | 29 (78.4%) |  |  |
| Has a healthcare professional (e.g. doctor/nurse) ever examined your testicles? | 74 |  |  | 0.7 (0.3 to 1.8) | 0.48 |
| No |  | 21 (56.8%) | 24 (64.9%) |  |  |
| Yes |  | 16 (43.2%) | 13 (35.1%) |  |  |
| *^1^* n (%) | | | | | |
| *^2^* Treatment effect estimated using logistic regression. | | | | | |

# **S6 Table.**Testicular self-examination behaviours at T2

| **Characteristic** | **N** | **E-MAT_E_**, N = 32*^1^* | **E-MAT_VR_**, N = 34*^1^* | **Odds ratio (95% CI)***^2^* | **p-value** |
| --- | --- | --- | --- | --- | --- |
| Have you advised at least one man (e.g., friend, colleague, partner, family member…) about the importance of feeling/ examining his testicles in the shower/bath at least once in the past three months? | 66 |  |  | 0.6 (0.2 to 1.8) | 0.39 |
| No |  | 9 (28.1%) | 13 (38.2%) |  |  |
| Yes |  | 23 (71.9%) | 21 (61.8%) |  |  |
| Have you purposefully felt/examined your testicles within the past three months? | 66 |  |  | NA | 1 |
| No |  | 3 (9.4%) | 0 (0%) |  |  |
| Yes |  | 29 (90.6%) | 34 (100%) |  |  |
| *^1^* n (%) | | | | | |
| *^2^* Treatment effect estimated using logistic regression. NA = OR can’t be estimated due to complete separation. | | | | | |

# **S7 Table.** Reliabilities for multi-item scales

| **Instrument** | **Timepoint** | **Internal consistency *^1^*** |
| --- | --- | --- |
| **Knowledge questionnaire** | T0 | 0.5 |
|  | T1 | 0.5 |
|  | T2 | 0.5 |
| **Testicular awareness** | T0 | 0.8 |
|  | T1 | 0.5 |
|  | T2 | 0.9 |
| **Implementation intentions** | T0 | 0.7 |
|  | T1 | 0.8 |
|  | T2 | 0.8 |
| **General Health Seeking Questionnaire** | T0 | 0.5 |
|  | T1 | 0.7 |
|  | T2 | 0.4 |

*^1^* Internal consistency measured by Cronbach’s alpha or the Kuder-Richardson Formula 20. Values range from 0-1, with 1 reflecting perfect consistency.

# **S8 Table.**Distribution of testicular awareness scores by trial arm and time point

| **Characteristic** | **N*^1^*** | **Overall**, N = 74*^2^* | **E-MAT_E_**, N = 37*^2^* | **E-MAT_VR_**, N = 37*^2^* |
| --- | --- | --- | --- | --- |
| Testicular Awareness Score at T0 | 74 | 3.6 (0.6) | 3.6 (0.5) | 3.6 (0.7) |
| Testicular Awareness Score at T1 | 74 | 4.0 (0.6) | 3.8 (0.6) | 4.2 (0.5) |
| Testicular Awareness Score at T2 | 66 | 4.1 (0.5) | 4.1 (0.4) | 4.0 (0.5) |
| *^1^* N=74 participants at T0 and T1 and 66 at T2 due to 8 participants lost to follow up.  *^2^* Mean (SD) | | | | |

# **S9 Table.** Perceived risk at by trial arm and time point

| **Characteristic** | **T0** | | | **T1** | | | **T2** | | |
| --- | --- | --- | --- | --- | --- | --- | --- | --- | --- |
|  | **N** | **E-MAT_E_**, N = 37*^1^* | **E-MAT_VR_**, N = 37*^1^* | **N** | **E-MAT_E_**, N = 37*^1^* | **E-MAT_VR_**, N = 37*^1^* | **N** | **E-MAT_E_**, N = 32*^1^* | **E-MAT_VR_**, N = 34*^1^* |
| **I am at risk for developing one or more testicular disease(s)** | 74 |  |  | 74 |  |  | 66 |  |  |
| Strongly Disagree |  | 0 (0%) | 1 (2.7%) |  | 1 (2.7%) | 1 (2.7%) |  | 1 (3.1%) | 0 (0%) |
| Disagree |  | 7 (18.9%) | 4 (10.8%) |  | 10 (27.0%) | 11 (29.7%) |  | 6 (18.8%) | 8 (23.5%) |
| Neither Agree nor Disagree |  | 15 (40.5%) | 23 (62.2%) |  | 12 (32%) | 14 (37.8%) |  | 10 (31.3%) | 14 (41.2%) |
| Agree |  | 14 (37.8%) | 9 (24.3%) |  | 14 (38%) | 9 (24.3%) |  | 14 (43.8%) | 12 (35.3%) |
| Strongly Agree |  | 1 (2.7%) | 0 (0%) |  | 0 (0%) | 2 (5.4%) |  | 1 (3.1%) | 0 (0%) |
| *^1^* n (%) | | | | | | | | | |

# **S10 Table.** Implementation intentions by trial arm and time point

| **Characteristic** | **T0** | | | **T1** | | | **T2** | | |
| --- | --- | --- | --- | --- | --- | --- | --- | --- | --- |
|  | **N** | **E-MAT_E_**, N = 37*^1^* | **E-MAT_VR_**, N = 37*^1^* | **N** | **E-MAT_E_**, N = 37*^1^* | **E-MAT_VR_**, N = 37*^1^* | **N** | **E-MAT_E_**, N = 32*^1^* | **E-MAT_VR_**, N = 34*^1^* |
| **I intend to feel my testicles in the shower/bath at least once over the coming month** | 74 |  |  | 74 |  |  | 66 |  |  |
| Strongly Disagree |  | 0 (0%) | 0 (0%) |  | 0 (0%) | 0 (0%) |  | 0 (0%) | 0 (0%) |
| Disagree |  | 2 (5.4%) | 2 (5.4%) |  | 0 (0%) | 0 (0%) |  | 0 (0%) | 1 (2.9%) |
| Neither Agree nor Disagree |  | 3 (8.1%) | 4 (10.8%) |  | 1 (2.7%) | 0 (0%) |  | 1 (3.1%) | 3 (8.8%) |
| Agree |  | 20 (54.1%) | 18 (48.6%) |  | 21 (56.8%) | 17 (45.9%) |  | 19 (59.4%) | 17 (50.0%) |
| Strongly Agree |  | 12 (32.4%) | 13 (35.1%) |  | 15 (41%) | 20 (54.1%) |  | 12 (37.5%) | 13 (38.2%) |
| **I will feel my testicles in the shower/bath at least once over the coming month** | 74 |  |  | 74 |  |  | 66 |  |  |
| Strongly Disagree |  | 0 (0%) | 0 (0%) |  | 0 (0%) | 0 (0%) |  | 0 (0%) | 1 (2.9%) |
| Disagree |  | 1 (2.7%) | 0 (0%) |  | 0 (0%) | 0 (0%) |  | 0 (0%) | 1 (2.9%) |
| Neither Agree nor Disagree |  | 6 (16%) | 3 (8.1%) |  | 0 (0%) | 0 (0%) |  | 2 (6.3%) | 2 (5.9%) |
| Agree |  | 17 (45.9%) | 22 (59.5%) |  | 21 (56.8%) | 16 (43.2%) |  | 19 (59.4%) | 16 (47.1%) |
| Strongly Agree |  | 13 (35.1%) | 12 (32.4%) |  | 16 (43.2%) | 21 (56.8%) |  | 11 (34.4%) | 14 (41.2%) |
| **I intend to advise at least one man (e.g. friend, colleague, partner, family member…) about the importance of examining his testicles in the shower/bath at least once in the coming month** | 74 |  |  | 74 |  |  | 66 |  |  |
| Strongly Disagree |  | 0 (0%) | 3 (8.1%) |  | 0 (0%) | 0 (0%) |  | 0 (0%) | 0 (0%) |
| Disagree |  | 6 (16.2%) | 2 (5.4%) |  | 5 (13.5%) | 0 (0%) |  | 2 (6.3%) | 4 (11.8%) |
| Neither Agree nor Disagree |  | 4 (10.8%) | 7 (18.9%) |  | 3 (8.1%) | 4 (10.8%) |  | 4 (12.5%) | 6 (17.6%) |
| Agree |  | 21 (56.8%) | 16 (43.2%) |  | 16 (43.2%) | 17 (45.9%) |  | 20 (62.5%) | 15 (44.1%) |
| Strongly Agree |  | 6 (16.2%) | 9 (24.3%) |  | 13 (35.1%) | 16 (43.2%) |  | 6 (18.8%) | 9 (26.5%) |
| *^1^* n (%) | | | | | | | | | |

# **S11 Table.** Distribution of General Help Seeking Questionnaire scores by trial arm and time point

| **Characteristic** | **N***^1^* | **Overall**, N = 74*^2^* | **Study Arm** | |
| --- | --- | --- | --- | --- |
|  |  |  | **E-MAT_E_**, N = 37*^2^* | **E-MAT_VR_**, N = 37*^2^* |
| General Help Seeking Questionnaire Enlargement at T0 | 74 | 3.3 (0.7) | 3.2 (0.6) | 3.4 (0.8) |
| General Help Seeking Questionnaire Lump at T0 | 74 | 3.0 (0.8) | 3.1 (0.6) | 3.0 (0.9) |
| General Help Seeking Questionnaire Pain at T0 | 74 | 3.0 (0.9) | 2.9 (0.7) | 3.0 (1.0) |
| General Help Seeking Questionnaire Enlargement at T1 | 74 | 3.3 (1.0) | 3.3 (0.8) | 3.3 (1.1) |
| General Help Seeking Questionnaire Lump at T1 | 74 | 3.2 (1.0) | 3.1 (0.8) | 3.2 (1.2) |
| General Help Seeking Questionnaire Pain at T1 | 74 | 3.2 (1.1) | 3.2 (0.8) | 3.3 (1.3) |
| General Help Seeking Questionnaire Enlargement at T2 | 66 | 3.3 (0.7) | 3.3 (0.6) | 3.4 (0.8) |
| General Help Seeking Questionnaire Lump at T2 | 66 | 3.2 (0.8) | 3.1 (0.7) | 3.3 (0.9) |
| General Help Seeking Questionnaire Pain at T2 | 66 | 3.2 (0.8) | 3.1 (0.7) | 3.3 (0.8) |
| *^1^* N=74 participants at T0 and T1 and 66 at T2 due to 8 participants lost to follow up.  *^2^* Mean (SD) | | | | |
