# Supplementary material for: Enhancing Men’s Awareness of Testicular Diseases (E-MAT) using virtual reality: A randomised pilot feasibility study and mixed method process evaluation
Source: PLoS One. 2024 Jul 22;19(7):e0307426. doi: 10.1371/journal.pone.0307426 (PMC11262699; doi:10.1371/journal.pone.0307426)
Supplement: Attachment — Submitted filename: Response to Reviewers.docx [file pone.0307426.s004.docx]

**Enhancing Men's Awareness of Testicular Diseases (E-MAT) Using Virtual Reality: A Randomised Pilot Feasibility Study and Mixed Method Process Evaluation**

We would like to thank the Editor and Reviewers of *PLOS ONE* for their valuable feedback regarding this manuscript. Below are our point-by-point responses to all comments. Changes in the text are made using track changes. Note that the line and page numbers below are provided for both the track changed copy of the manuscript and the clean copy.

| **JOURNAL REQUIREMENTS** | | | |
| --- | --- | --- | --- |
| **Comment** | **Our Response** | **Page and line number *in marked document*** | **Page and line number *in clean copy*** |
| 1. Please ensure that your manuscript meets PLOS ONE's style requirements, including those for file naming. The PLOS ONE style templates can be found at *[two links provided, too long to include here]* | We ensured that our manuscript meets all PLOS ONE style requirements. | Throughout | Throughout |
| 2. We suggest you thoroughly copyedit your manuscript for language usage, spelling, and grammar. If you do not know anyone who can help you do this, you may wish to consider employing a professional scientific editing service. | This has been done among the authors who are fluent and native English speakers and seasoned academics. | Throughout | Throughout |
| 3. Thank you for stating the following financial disclosure:  "Health Research Board Definitive Interventions and Feasibility Awards (DIFA-2020-028)."  Please state what role the funders took in the study. If the funders had no role, please state: ""The funders had no role in study design, data collection and analysis, decision to publish, or preparation of the manuscript.""  If this statement is not correct you must amend it as needed.  Please include this amended Role of Funder statement in your cover letter; we will change the online submission form on your behalf. | The role of the funders was stated in the cover letter of the original submission. We included this statement again in the cover letter for the revised manuscript. | Cover Letter | Cover Letter |
| 4. Please provide a complete Data Availability Statement in the submission form, ensuring you include all necessary access information or a reason for why you are unable to make your data freely accessible. If your research concerns only data provided within your submission, please write "All data are in the manuscript and/or supporting information files" as your Data Availability Statement. | The data that support the findings of this study are openly available in the Open Science Foundation at <https://osf.io/m5wb7/>. | Submission form | Submission form |
| 5. We note that you have referenced (Eldridge SM, Lancaster GA, Campbell MJ, Thabane L, Hopewell S, Coleman CL, et al. Defining Feasibility and Pilot Studies in Preparation for Randomised Controlled Trials: Development of a Conceptual Framework. Lazzeri C, editor. PLOS ONE. 2016 Mar 15;11(3):e0150205.) which has currently not yet been accepted for publication. Please remove this from your References and amend this to state in the body of your manuscript: (ie “Bewick et al. [Unpublished]”) as detailed online in our guide for authors  http://journals.plos.org/plosone/s/submission-guidelines#loc-reference-style | We have discussed this with the Senior Staff Editor Miquel Vall-llosera Camps who, and I quote, *“discussed this with the Senior Editorial staff and we agree that that reference was published, so our recommendation is to proceed as you suggest and keep that reference. When you resubmit please include in your response to reviewers your explanation for using this reference, as provided here.”*  To this end, the reference to Eldrige et al.’s paper will remain as is. | Throughout | Throughout |
| 6. We note that Figure 3 in your submission contain copyrighted images. All PLOS content is published under the Creative Commons Attribution License (CC BY 4.0), which means that the manuscript, images, and Supporting Information files will be freely available online, and any third party is permitted to access, download, copy, distribute, and use these materials in any way, even commercially, with proper attribution. For more information, see our copyright guidelines: http://journals.plos.org/plosone/s/licenses-and-copyright. | We have discussed this with Teresa Diviacchi, Peer Review Operations Specialist. To this end, for Figure 3, I confirm that the screenshots shown are from the E-MAT_VR_ intervention and that I hold the copyright for this educational tool. I have submitted the Content Permission Form accordingly. | Content Permission Form | Content Permission Form |
| 7. Please include captions for your Supporting Information files at the end of your manuscript, and update any in-text citations to match accordingly. Please see our Supporting Information guidelines for more information: http://journals.plos.org/plosone/s/supporting-information. | Captions for Supporting Information files included as recommended and references to these files in the text of the manuscript have been updated accordingly. | Page 47  Lines 1-19 | Page 44  Lines 1-19 |
| 8. Please review your reference list to ensure that it is complete and correct. If you have cited papers that have been retracted, please include the rationale for doing so in the manuscript text, or remove these references and replace them with relevant current references. Any changes to the reference list should be mentioned in the rebuttal letter that accompanies your revised manuscript. If you need to cite a retracted article, indicate the article’s retracted status in the References list and also include a citation and full reference for the retraction notice. | Reference list revised as recommended. | Throughout | Throughout |

| **EDITOR** | | | |
| --- | --- | --- | --- |
| **Comment** | **Our Response** | **Page and line number *in marked document*** | **Page and line number *in clean copy*** |
| To facilitate the review process and enhance communication between you and the reviewers, we strongly recommend adding line numbers to your manuscript. | Line numbers added as recommended. | Throughout | Throughout |
| Furthermore, it has been noted by two of our reviewers that there appear to be some discrepancies in your report (comment 10 by Reviewer 5 and comment 14 by Reviewer 1). We kindly ask that you address these issues and provide an explanation for them in your revised submission. | All reviewers’ comments including comment 10 by Reviewer 5 and comment 14 by Reviewer 1 have been addressed and all discrepancies have been amended/resolved. | Response to Reviewers 1 and 5 | Response to Reviewers 1 and 5 |
| Lastly, we would like to draw your attention to the 7th comment made by Reviewer 5. This particular point requires your special attention during the revision process. | Thank you for highlighting this comment which we have addressed in full. Please see our response to Reviewer 5 below. | Response to Reviewer 5 | Response to Reviewer 5 |

| **REVIEWER 1** | | | |
| --- | --- | --- | --- |
| **Comment** | **Our Response** | **Page and line number *in marked document*** | **Page and line number *in clean copy*** |
| The title could be improved | Shortened as follows: Enhancing Men's Awareness of Testicular Diseases (E-MAT) Using Virtual Reality: A Randomised Pilot Feasibility Study and Mixed Method Process Evaluation | Page 1  Lines 1-3 | Page 1  Lines 1-3 |
| For the exclusion criteria, other criteria are to be stated. | In addition to excluding individuals with a history of seizures and motion sickness, we added the following exclusion criteria as suggested: Individuals who were younger than 18 years or older than 50 years, not assigned male at birth, residing outside the Republic of Ireland, and not involved in GAA as either players or coaches were excluded. | Page 11  Lines 1-3 | Page 10  Lines 8-10 |
| The following requires further information.   i) Page 10, the person who collected the info 'T0 and T1 data were collected in person in participants’ respective GAA clubs' is to be stated. ii) Page 10, the person who performs the randomization. iii) Blinding information whether just involving the participants and data analyst. | i) Four research personnel collected T0 and T1 data in-person in participants’ respective GAA clubs. ii) For each participating club, individual participants were randomised with the same probability to one of the two trial arms (E-MAT_VR_ or E-MAT_E_) using the Castor EDC software. See new section titled “Randomisation” for the full process. iii) We have provided information regarding blinding in the original submission. However, to ensure clarity and in accordance with CONSORT, we added a new section titled “Randomisation” where we explained randomisation as well as blinding. Given the differences between the two arms, participants were made aware of the arm they were randomised to once they were assigned to either E-MAT_VR_ or E-MAT_E_. Outcome assessments were self-reported and returned to research staff with no information on allocation. The data analyst was also blinded to allocation until database lock and completion of the protocol-specified data analysis. | Page 13  Lines 2,3  Page 22  Lines 2-8  Page 22  Lines 9-13 | Page 11  Lines 17,18  Page 19  Lines 22,23  Page 20  Lines 6-10 |
| Information on the pilot testing of the researcher-designed instruments prior to their usage is to be described. | All instruments used in the pilot feasibility trial and most instruments used in the process evaluation were developed, validated, and pilot tested elsewhere [references provided]  As for instruments developed purposefully for this study: interview guides were developed by four senior academics and researchers who have experience in qualitative research (MMS, MMC, MPD, JH).  As for fidelity checklists, these were purposefully developed for use in the current study. Checklist items were mapped onto the processes and contents of the E-MAT_VR_ intervention and E-MAT_E_ control. These checklists were pilot tested with four participants. Data from these participants were not included in the final analysis. No changes were made to these checklists following pilot testing.  Finally, the time it took participants to complete E-MAT_VR_ (in minutes) was captured automatically in the VR headset and the time it took participants to complete E-MAT_E_ (in minutes) was captured manually by the observing researcher. | Page 15  Lines 5-8  Page 18  Lines 5-7  Page 18  Lines 25,26  Page 19  Lines 1-3  Page 19  Lines 6-8 | Page 13  Lines 20-24  Page 15  Lines 5-7  Page 16  Lines 26,27  Page 17  Line 1  Page 17  Lines 2-6 |
| The information for the sample size is unclear. More elaboration for the statement ‘detect failures in study processes that occur just 5% of the time’ is to be provided. The primary outcome, effect size, power, significance level, expected proportion of participants experiencing the outcome, sample size prior the consideration of attrition rates is to be stated | The goal of a pilot feasibility study is to identify problems that would impede the conduct of a larger, definitive RCT as opposed to establishing the efficacy of an intervention. There is no gold standard for sample size calculation in pilot feasibility studies and sample sizes as small as 10 and as large as 59 have been recommended (Saab et al., 2018). Julious (2005), for example, suggested a sample size of 12 per arm, whereas Viechtbauer et al. (2015) proposed a formula to calculate the sample size in pilot studies and reported that “if a problem exists with 5% probability in a potential study participant, the problem will almost certainly be identified (with 95% confidence) in a pilot study including 59 participants” (p. 1375). Therefore, we set the sample size at 59 in our study. Allowing for a potential attrition rate of 25%, we aimed to recruit 74 participants. | Page 21  Lines 11-21 | Page 19  Lines 11-20 |
| For the statement ‘’Qualitative data analysis was conducted by one researcher and checked for accuracy by two researchers to minimise errors and improve data credibility and confirmability’ is the researcher(s)referring to the authors of this study? | Indeed, we clarified that qualitative data analysis was conducted by one author (MMC) and checked for accuracy by two authors (MPD, JH) to minimise errors and improve data credibility and confirmability. | Page 23  Lines 18,19 | Page 21  Lines 13-15 |
| Page 19, the analysis of whether intent to treat or per protocol is to be stated. | We did estimate between-arm contrasts for the knowledge scores and the testicular self-examination behaviours on an intent-to-treat basis | Page 22  Line 19 | Page 20  Lines 14-16 |
| Information on handling missing (if any) is to be stated. | Missing data were addressed using complete case analysis, following from the small number of missing values. | Page 22  Lines 22,23 | Page 20  Lines 19,20 |
| Table 2, the decimal point for percentage figure is to be standardized e.g. 1 decimal point. Likewise in the text and the supplementary tables. | All decimal points in the text and tables including supplemental tables are now at 1 decimal point. | Table 2 and throughout | Table 2 and throughout |
| Page 20, the number of participants who participated in the interview/Focus Group with the researchers for the qualitative study is to be stated. | All participants were invited to participate in an individual interview with one of the authors (MMC) as part of the process evaluation. Of those, a sub-sample of nine participants agreed to participate (six from E-MAT_E_ and three from E-MAT_VR_). Eight interviews were conducted face-to-face and one by telephone. Interviews with participants lasted on average five minutes. Finally, all four researchers who collected data participated in a one-hour virtual focus group that was facilitated by the lead author (MMS). | Page 25  Lines 1-6 | Page 22  Lines 18-23 |
| Page 23, the statement Thirty-six (97%) participants in both E-MATVR and E-MATE either agreed or strongly agreed that the intervention' does not reflect figure(s) in Supplementary Table S2. | Thank you and apologies for this error. We have now combined Tables S2 and S3 since data in both tables are form the same instrument. We renumbered all supplementary tables accordingly. | Supporting Table S2 | Supporting Table S2 |
| For the supplementary tables, ensure the decimal points are standardized and to double-check the p values. | All decimal points have now been reduced to 1 decimal point and all p-values have been double checked and reported in full. | Throughout | Throughout |
| Supplementary Table S2, S4, symbol > for p value to be avoided. Actual p value is to be presented. | Actual p-value stated as recommended. | Tables S2 and S3 (formerly S4) | Tables S2 and S3 (formerly S4) |
| Supplementary Table S8, the decimal point for internal consistency is to be reduced. | Reduced to 1 decimal point. This is now Table S7. | Table S7 | Table S7 |
| Table 5, Supplementary Table S9, S12, the discrepancy n=66 and n=74 at T2 is to be denoted in the table footnote. | “N=74 participants at T0 and T1 and 66 at T2 due to 8 participants lost to follow up.” This explanation was added as footnote in Tables 5, S8 (was S9), and S11 (was S12). | Table 5  Table S8  Table S11 | Table 5  Table S8  Table S11 |
| Ensure all the statistical tests used in the results section are mentioned in the statistical methods in page 19. | Simple between-arm tests for ordinal responses were conducted using Kruskal-Wallis rank sum tests. | Page 23  Lines 2,3 | Page 20  Lines 23,24 |
| For Fisher’s exact test, 1 or 2-tailed test is to be stated. | All reported p-values are based on two-sided tests with statistical significance defined as p < 0.05.  Simple between-arm tests for ordinal responses were conducted using Kruskal-Wallis rank sum tests. | Page 23  Lines 2-6 | Page 20  Lines 23-25  Page 21  Lines 1,2 |
| The accepted level of statistical significance (and 1 or 2-tailed test) is to be stated. | All reported p-values are based on two-sided tests with statistical significance defined as p<0.05 | Page 23  Lines 5,6 | Page 21  Lines 1,2 |
| Ensure all information that were reported in the CONSORT checklist are presented/clearly presented in the manuscript. | As recommended, we made sure that all information in the CONSORT checklist is clearly presented in the manuscript. We changed some of the headings/sub-headings accordingly. | Throughout | Throughout |
| The numbering for each subtopic/subtitle is to be omitted. | Omitted as recommended. | Throughout | Throughout |

| **REVIEWER 2** | | | |
| --- | --- | --- | --- |
| **Comment** | **Our Response** | **Page and line number *in marked document*** | **Page and line number *in clean copy*** |
| 1-I recommend to revise the keywords ; as the "sport" isn't appropriate keyword. | Thank you for your comment. We deleted the keyword “Sport” and replaced it with “Athletes” to reflect our target population. | Page 5  Line 16 | Page 5  Line 12 |
| 2-The golden time of 6 hours for testicular torsion is not the rule , and this phrase is a little misleading , it depends on degree of torsion , I think it should be replaced with most recent literature | Thank you. We replaced this statement with the following: “Testicular torsion requires aggressive management in men presenting with testicular pain that has been ongoing for many hours, even 24 hours or more from the onset of ischemia.” Here, we cited [this systematic review](https://pubmed.ncbi.nlm.nih.gov/28953100/). | Page 7  Lines 9-13 | Page 6  Lines 20-24 |
| 3-I suggest to include all meaningful data in Abstract. | The abstract has been reviewed as per your recommendation and recommendation by Reviewer 5. It is now structured and has more meaningful data. | Abstract Pages 4 and 5 | Abstract Pages 4 and 5 |
| 4-I recommend to summarize or merge findings to better presentation of work, it seems the findings reported several times in text. | Thank you for your comment. We reviewed the results and noted that the main results are presented in the text of our manuscript without duplication and in line with the outcomes listed under “Methods.” The remainder is presented in the tables inclusive of supplemental tables.  As for merging results, we were able to merge findings from open-ended questions and findings from qualitative interviews with participants. | Pages 27 and 28 | Page 25 and 26 |

| **REVIEWER 3** | | | |
| --- | --- | --- | --- |
| **Comment** | **Our Response** | **Page and line number *in marked document*** | **Page and line number *in clean copy*** |
| Points 1-9 | Thank you so much your positive comments and insights regarding our paper. We are delighted to hear that our study provides a solid foundation for a future RCT. | N/A | N/A |
| 1. Explore strategies to further enhance the accessibility and user experience of the VR intervention, addressing the challenges identified in this pilot study. | Thank you for your comment. Based on your recommendation and recommendations by Reviewer 5, we included a new section within the discussion where we discussed the strengths, limitations, and lessons learned for a future RCT. Here, we critically discussed strategies to enhance accessibility while addressing the challenges to same. | Section titled “Strengths, limitations, and lessons learned” from Page 42 to 46 | Section titled “Strengths, limitations, and lessons learned” from Page 39 to 43 |
| 2. Consider incorporating more interactive elements and gamification features to engage participants and potentially improve the effectiveness of the interventions. | Thank you for your comment. This has now been included under “Lesson 3.” | Page 44 Lines 3-8 | Page 41  Lines 10-15 |
| 3. Carefully examine the survey length and content to ensure optimal participant engagement and data collection. | Thank you for your comment. This has now been included under “Lesson 8.” | Page 45  Lines 17-23 | Page 43  Lines 1-7 |
| 4. Expand the study population to include a more diverse sample of men, potentially beyond the current focus on Gaelic game athletes. | Thank you for your comment. This has now been included under “Lesson 5.” | Page 44  Lines 22-26  Page 45  Lines 1-3 | Page 42  Lines 3-10 |

| **REVIEWER 4** | | | |
| --- | --- | --- | --- |
| **Comment** | **Our Response** | **Page and line number *in marked document*** | **Page and line number *in clean copy*** |
| The study is very sound and well written. I have no issues with the premises, nor with the content. There is good recruitment and followup, and I like the addition of a couple of paragraphs at the start of the discussion regarding successful recruitment. | Thank you so much for your positive comments and insights regarding our paper. We are delighted to hear that our discussion around successful recruitment is of value. | N/A | N/A |
| My only comment would be that there does not seem to be any real difference in efficacy between the two arms, suggesting that the use of VR is not necessarily an improvement over more conventional interventions. It would be nice to see this commented on in more detail in the discussion. In particular, the assertion that VR may 'ensure access to hard to reach people and places' and is an 'alternative opportunity in delivering health promoting information to hard to reach populations' would seem counterintuitive, given the additional technology burden and distribution issues with VR. I think these statements would benefit from more consideration and explanation. | Thank you for your comment. We added a list of lessons learned from our trial based on this comment and comments made by the other Reviewers.  One of the lessons relates to using VR to promote the health of young men. Here, we provided figures on the market size of VR and the profile of users who are mainly male aged 18 to 54 years. We also explained that the pilot feasibility study outcomes improved for all participants but did not differ by arm, which is mainly because our trial, by design, was not sufficiently powered and was geared towards feasibility rather than efficacy. We then concluded that the conduct of a future RCT is needed to test the efficacy of the intervention. We also included two lessons learned in relation to the importance of estimating resources incurred by the intervention and the importance of revisiting and refining the intervention. | Section titled “Strengths, limitations, and lessons learned” from Page 42 to 46 | Section titled “Strengths, limitations, and lessons learned” from Page 39 to 43 |

| **REVIEWER 5** | | | |
| --- | --- | --- | --- |
| **Comment** | **Our Response** | **Page and line number *in marked document*** | **Page and line number *in clean copy*** |
| 1- Please add line numbering to your manuscript to enhance communications. | Added as recommended. | Throughout | Throughout |
| 2- The sentence "challenges related to getting used to VR, nausea, and technical issues." is a fragment of a sentence and should be edited. | Sentence rephrased as follows: “Initial difficulty using VR equipment, nausea, and technical issues were identified as challenges to engaging with E-MAT_VR_” | Page 4  Lines 25,26 | Page 4  Lines 24,25 |
| 3- A structured abstract would be a better fit for your study. I recommend sectioning your abstract in a way that helps the reader to grasp the essence of your report. | The abstract is now structured as recommended. | Pages 4 and 5 in full | Pages 4 and 5 in full |
| 4- The abstract's conclusion is too brief and does not fully communicate the limitations, interpretations, etc. Please trim it and make room for more necessary information. | We reworked the abstract, including the conclusion section and highlighted the key implications/ recommendations from our study. | Page 5  Lines 8-14 | Page 5  Lines 6-10 |
| 5- A good proportion of your introduction is devoted to the clinical and epidemiological aspects of the diseases. I think focusing more on details directly related to your topic will greatly enhance the readability and relevance of your study.  I suggest to discuss the rationale for the future RCT, the reasons to justify why the pilot trial is needed, and areas of uncertainty that need to be addressed before the future RCT can take place, in depth at the end of your introduction. | Thank you for your comment. We shorted the clinical and epidemiological details while being cognisant of comments made by other reviewers, particularly Reviewer 2 who requested the inclusion of recent citations in relation to testicular torsion.  As for your recommendation to discuss the rationale for a future RCT, we added a section at the end of the introduction explaining previous pilot work that we have completed, providing a rationale for the current study in the context of feasibility trials and process evaluations, and explaining the uncertainties that we aimed to explore in our study ahead of conducting a future RCT. | Pages 6-9  Page 8  Lines 14-20 and 24-27  Page 9  Lines 1-13 | Pages 6-8  Page 7  Lines 18-27  Page 8  Lines 1-20 |
| 6- The ending statement of the introduction's second paragraph:  Your statement comes from a late reference (2014), while currently, there are evidence suggesting watchful observation (aka active surveillance) for testicular cancer. Please update your citation. | Thank you. We deleted this citation and inserted a short section on watchful surveillance with more recent citations. | Page 6  Lines 11-17 | Page 6  Lines 12-16 |
| 7- The study has limited the exclusion criteria to ones with seizure and/or motion sickness. However, in my opinion, a very important characteristic that can affect and potentially, confound the results is having prior encounters with testicular diseases in the person being studied or his close relatives and acquaintances. Such individuals are reasonably and naturally more familiar with clinical manifestations, burdens, treatments and overall experience of a patient with testicular diseases. Furthermore,most of the participants enrolled in the study are players and coaches in the GAA clubs that are more susceptible to testicular injuries and consequent diseases and might be more aware of testicular diseases. The mentioned points might affect not only the main outcomes but also the dose exposure, reach, and internal validity. It may be beneficial to provide justification for not considering such information at recruitment or mention it as a limitation to your study. | Thank you for your comment. This is indeed something that we have discussed among ourselves while designing this study. Given that the focus of our study was on process and feasibility rather than efficacy, we did not exclude participants with a history of testicular disease. We made this clear in the methods under “Eligibility criteria.”  We do acknowledge, however, that a history of testicular disease coupled with the fact that participants were GAA players and coaches who are susceptible to testicular injury and disease, might be limitation of our study (despite only having 2 of 74 participants disclose a personal history of a testicular disease). We elaborated on this potential confounder and mentioned the importance of considering it in the design of a future RCT. We also restructured the limitations section to incorporate lessons learned, one of these lessons (Lesson 4) calls for accounting for confounders in a future RCT. | Page 10  Lines 24-26  Page 44  Lines 9-21 | Page 10  Lines 5-7  Page 41  Lines 16-26  Page 42  Lines 1,2 |
| 8- The study compares an interactive (VR) VS a non-interactive (PDF document) method of education. I assume the first question that comes to the mind of readers (providers and policymakers) is whether VR, which is an expensive technology, can outperform other interactive methods and whether it is cost-effective for them to adopt in in their system. Nonetheless, it is well-known that using interactive models is usually more effective than non-interactive ones. It would be helpful if the authors provide explanations for this point or include it as a limitation of the study. | Thank you for your comment. Based on your recommendation and recommendations by Reviewer 3, we included a new section within the discussion where we discussed the strengths, limitations, and lessons learned for a future RCT. One of the lessons learned (Lesson 2) relates to the cost of technology, resources incurred in the study, and future plan for an economic evaluation. | Page 43  Lines 13-23 | Page 40  Lines 21-26  Page 41  Lines 1-9 |
| 9- I see the authors provide a lot of supporting information. While this abundance is a good and encouraged practice, I recommend gathering them into a single file with an informative table of contents to facilitate the readers experience. Downloading that many files could be tedious. | Thank you. We welcome this recommendation as long as the journal allows it. We have now combined all supplementary tables into one document. We included a table of contents at the start of this document to signpost the readers. We also merged Supplementary Tables S2 and S3 since they are part of the same instrument and relabeled all supplementary tables accordingly. We now have 11 supplementary tables. | Supplementary Tables | Supplementary Tables |
| 10- Table 2 contains redundant information regarding the gender combination of the participants. | The gender row has now been deleted since all the included participants were male-identifying. | Table 2 on Pages 25,26 | Table 2 on Page 23 |
| 11- On page 24, part 3.2.5, the information mentioned in the sentence, "Elements of the VR game that participants did not favor ..." is not the same as those in the supplementary table S5. | Thank you for spotting this inaccuracy. Our apologies. We reviewed this section and made sure that there are no discrepancies between the text of the manuscript and Table S5 (now Table S4). | Page 28  Lines 1-10 | Page 25  Lines 16-26 |
| 12- The discussion could benefit from explaining the limitations in detail and arguing whether and how they could be overcome in the future RCT. | We have edited the discussion and, as aforementioned, included a new section where we discussed the strengths, limitations, and lessons learned for a future RCT. | Section titled “Strengths, limitations, and lessons learned” from Page 42 to 46 | Section titled “Strengths, limitations, and lessons learned” from Page 39 to 43 |
| In summary, your article makes a valuable contribution to the field, and I believe the aforementioned suggestions will further enhance it. | Thank you for your positive comment. | N/A | N/A |
